# TRP Channels as Emerging Therapeutic Targets for Neurodegenerative Diseases

**DOI:** 10.3389/fphys.2020.00238

**Published:** 2020-04-15

**Authors:** Chansik Hong, Byeongseok Jeong, Hyung Joon Park, Ji Yeon Chung, Jung Eun Lee, Jinsung Kim, Young-Cheul Shin, Insuk So

**Affiliations:** ^1^Department of Physiology, Chosun University School of Medicine, Gwangju, South Korea; ^2^Department of Neurology, Chosun University School of Medicine, Gwangju, South Korea; ^3^Department of Physiology and Institute of Dermatological Science, Seoul National University College of Medicine, Seoul, South Korea; ^4^Department of Cell Biology, Harvard Medical School, Boston, MA, United States

**Keywords:** TRP, Ca^2+^, ROS, cell death, neurodegeneration, AD, PD, HD

## Abstract

The development of treatment for neurodegenerative diseases (NDs) such as Alzheimer’s disease, Parkinson’s disease, Huntington’s disease, and amyotrophic lateral sclerosis is facing medical challenges due to the increasingly aging population. However, some pharmaceutical companies have ceased the development of therapeutics for NDs, and no new treatments for NDs have been established during the last decade. The relationship between ND pathogenesis and risk factors has not been completely elucidated. Herein, we review the potential involvement of transient receptor potential (TRP) channels in NDs, where oxidative stress and disrupted Ca^2+^ homeostasis consequently lead to neuronal apoptosis. Reactive oxygen species (ROS) -sensitive TRP channels can be key risk factors as polymodal sensors, since progressive late onset with secondary pathological damage after initial toxic insult is one of the typical characteristics of NDs. Recent evidence indicates that the dysregulation of TRP channels is a missing link between disruption of Ca^2+^ homeostasis and neuronal loss in NDs. In this review, we discuss the latest findings regarding TRP channels to provide insights into the research and quests for alternative therapeutic candidates for NDs. As the structures of TRP channels have recently been revealed by cryo-electron microscopy, it is necessary to develop new TRP channel antagonists and reevaluate existing drugs.

## Introduction

Neurodegenerative disorders (NDs) are one of the most devastating types of chronic diseases and lead to a significant social and medical burden on society. With the growing elderly population, the number of patients with NDs is also increasing. Although many pharmaceutical companies are struggling to develop novel therapeutics for neurological diseases, some of the world’s leading pharmaceutical companies have declared their abandonment of the development of therapeutics for NDs. Alzheimer’s disease (AD) is the most common ND, which accounts for 60–70% of all dementia (Association, 2016). Nevertheless, no new treatments for AD have been developed in over a decade. Some of the reasons for the difficulty in treating NDs are the combination of complex causative factors and irreversible structural and functional damage of neurons.

Neurodegenerative disorders are typically progressive, late-onset disorders, and aging is the greatest risk factor (Wakabayashi et al., 2014). In addition, genetic and environmental factors not only contribute to their pathogenesis independently but also interact with each other to increase their effects. The pathogenesis of NDs involves an initial toxic insult and consequences of the secondary pathological damage. The most primary causative hypothesis of AD is the intraneuronal accumulation of amyloid-beta (Aβ) and hyperphosphorylated tau protein (Iqbal et al., 2010; Murphy and LeVine, 2010). Parkinson’s disease (PD) is caused by the degeneration of dopaminergic neurons in the substantia nigra par compacta (SNpc), with subsequent dopamine deficiency (Michel et al., 2013; Kalia and Lang, 2015). A type of hereditary ND, Huntington’s disease (HD) is an autosomal dominant disorder caused by CAG repeat expansion within the Huntington (*HTT*) gene (Kremer et al., 1994). The third common ND after AD and PD is amyotrophic lateral sclerosis (ALS) that is characterized by the deterioration of motor neurons.

Neurodegenerative disorders such as AD, PD, HD, and ALS are distinguished by clinical symptoms and specific neuronal sites with distinct pathology. However, apparent clinical symptoms are manifested only after extensive pathological damage, with significant neuronal and synaptic loss. Eventually, the contribution of individual insults reaches a common end state, which causes severe impairments in the function and plasticity of neuronal and glial cells (Rasband, 2016). Over the past few decades, there has been considerable effort to understand the pathogenesis of NDs. To date, a number of studies have reported that oxidative stress, ER (endoplasmic reticulum) stress, abnormal Ca^2+^ homeostasis, protein misfolding, aggregation, neuroinflammation, and mitochondrial dysfunction are highly related to neuronal damage. The relationship between them, however, has not been completely elucidated. Besides, even though Aβ is still a compelling candidate in the pathogenesis of AD, the latest experiments raise doubts about the Aβ hypothesis and Aβ -based drug development for AD (Du et al., 2018). Therefore, it is necessary to find the missing links amongst the risk factors of NDs and to discover new therapeutic targets based on novel mechanisms.

Ion channels are key determinants of brain function, since the physiological function of neurons is to carry information or impulses via electrical signals (action potentials) to communicate with each other at synapses. Thus, neurological channelopathies have been identified mainly in voltage-gated and ligand-gated channels or receptors that result from genetically determined defects in their function. However, based on patients with progressive NDs with adulthood manifestations, we have focused on the age-related susceptibility to environmental toxins and chemicals. Recently, emerging evidence has indicated that transient receptor potential (TRP) channels, ubiquitously expressed throughout the brain (Sawamura et al., 2017), play a significant role in the regulation of physiological functions, as well as in reactive oxygen species (RO)-related human diseases. Based on the polymodal activation of TRP channels acting as cellular sensors, many researchers are investigating their activation mechanisms (Takada et al., 2013). Here, we review the potential involvement of TRP channels in NDs, where oxidative stress and disrupted Ca^2+^ homeostasis have been characterized with respect to pathological consequences in neuronal apoptosis. Second, we discuss the latest findings in the field of TRP to provide insight into the research and quest for alternative therapeutic candidates for the treatment of NDs.

## The Critical Role of Ca^2+^ in the Pathogenesis of Neurodegenerative Diseases

Ca^2+^ homeostasis is crucial to the normal physiological functions of neurons, such as neuronal survival, growth, and differentiation. Hence, long-lasting Ca^2+^ dyshomeostasis can eventually lead to neuronal loss. Accumulated evidence strongly implicates that abnormal Ca^2+^ levels stimulate dysregulation of intracellular signaling, which consequently induces neuronal cell death (Barnham et al., 2004). Therefore, disruption of Ca^2+^ homeostasis in neuronal cells leads to ROS generation and ATP depletion, following the mitochondrial dysfunction in NDs such as AD, PD, HD, and ALS. Interestingly, a close correlation between the increase in [Ca^2+^]_i_ and other pathogenic mechanisms has been reported, such as Aβ deposition (Demuro et al., 2010), imbalance between ROS and antioxidant function (Gorlach et al., 2015), and mitochondrial dysfunction (Contreras et al., 2010; Pivovarova and Andrews, 2010).

Some reports have shown bidirectional crosstalk between amyloid pathology and the Ca^2+^ pathway. Most studies reported that Aβ increases intracellular Ca^2+^ levels by inducing ER Ca^2+^ depletion (Suen et al., 2003; Abramov et al., 2004b; Ferreiro et al., 2008). Abramov et al. (2004a) identified that Aβ causes Ca^2+^-dependent oxidative stress by the activation of NADPH oxidase in astrocytes and that the reduced antioxidant activity induces neuronal death. Recently, Calvo-Rodriguez et al. (2019) suggested that Aβ oligomers exacerbate Ca^2+^ remodeling from ER to mitochondria in aged neurons but not in young neurons. Conversely, Itkin et al. (2011) argued that Ca^2+^ stimulates the formation of Aβ oligomers, leading to neuronal toxicity in AD.

The imbalance between ROS production and antioxidant defenses results in the excessive accumulation of ROS and oxidative stress. Since aged neurons with low antioxidant capacity are more vulnerable to oxidative insults (Chen et al., 2012), ROS overproduction can chronically lead to irreversible oxidation (Ivanova et al., 2016). Oxidative stress also causes mitochondrial dysfunction, which itself aggravates ROS generation. Moreover, the opening of the mitochondrial permeability transition pore (mPTP) and the release of cytochrome c into cytoplasm activate pro-apoptotic caspases (Guo et al., 2013). Oxidative stress and mitochondrial dysfunction are well known to be related to an increase in cytosolic Ca^2+^ levels that underlies the pathogenesis of AD (Cenini and Voos, 2019), PD (Blesa et al., 2015), HD (Zheng et al., 2018), and ALS (Carri et al., 2015). Interestingly, the mitochondrial metabolic state can affect the Mg^2+^ concentration of both the matrix and the cytoplasm, where Mg^2+^ interferes with mitochondrial Ca^2+^ transport and mitochondrial ATP generation (Llorente-Folch et al., 2015). Since the efficient removal of [Ca^2+^]_i_ requires ATP, impairment of mitochondrial ATP generation prevents Ca^2 +^ pumps from operating both in the plasma membrane and in the ER (Ott et al., 2007). Thus, dysregulation of Ca^2+^ signaling is one of the key processes in early stage neuronal loss. In spite of its significance, the mediator of such aberrant Ca^2+^ increase and its source are not fully understood. To test the effectiveness of preventing Ca^2+^ overload for ND therapy, a variety of appropriate channel candidates should be further examined by developing channel-specific drugs for new channel targets. Last but not least, experimental data on existing drugs also need to be reevaluated.

Oxidative stress can directly modulate the gating properties of ion channel proteins. Pathological mechanisms underlying the dysregulation of ion channels by oxidation have been previously proposed in a variety of diseases, especially cancer (Reczek and Chandel, 2018) and NDs (Gorlach et al., 2015). Under normal conditions, defensive antioxidants can protect or repair the damage caused by oxidation. However, the target proteins are retained in their oxidized forms and are activated as long as the antioxidant activity is reduced. To date, several studies have shown that oxidative stress is involved in the modulation of activities of voltage-gated Ca^2+^ (Gorlach et al., 2015; Ramirez et al., 2016), Na^+^, and K^+^ (Sesti et al., 2010) channels and ligand-gated receptors such as NMDA (Kamat et al., 2016), AMPA (Joshi et al., 2015), GABA (Bradley and Steinert, 2016), and RyR (Zima and Mazurek, 2016). However, there is limited evidence that directly identifies the oxidative modification of a channel protein based on molecular mechanisms.

Since TRP channels are non-selective, Ca^2+^-permeable channels that can be opened at resting membrane potential in response to various stimuli, we focused on TRP channels. The activation of TRP channels consequently changes membrane depolarization toward the action potential threshold. When TRP channels open, they allow sodium and calcium into the cytoplasm, which subsequently triggers the opening of voltage-dependent Ca^2+^ channels. This is why TRP channels are upstream risk factors, ahead of voltage-dependent channels (Numata et al., 2011). Therefore, the hyperactivation of TRP channels is responsible for neuronal excitotoxicity, which is closely associated with NDs. In the following section, we will address the physiological and pathological roles of TRP channels in neurons through the recent studies related to TRP channels and our study of the TRPC5 channel.

## The Physiological and Pathological Role of TRP Channels in Neurons

**FIGURE 1:**
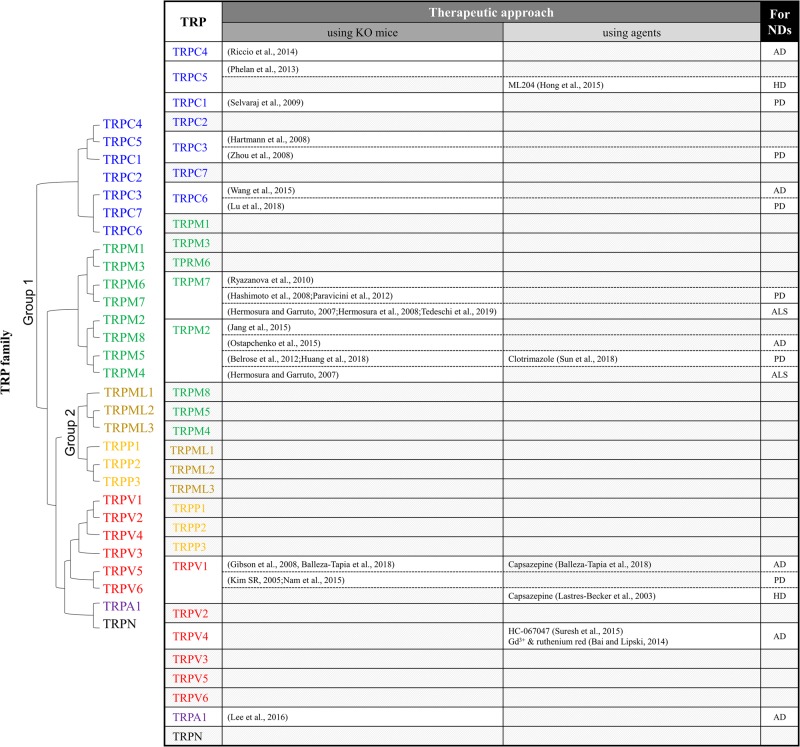
Summary of TRP studies using knockout mice or antagonists to investigate therapeutic targets of neurodegenerative diseases. ND, neurodegenerative diseases; AD, Alzheimer’s disease; PD, Parkinson’s disease; HD, Huntington’s disease; ALS, amyotrophic lateral sclerosis.

As mentioned above, TRP channels are widely expressed in almost every mammalian cell, predominantly in the brain. TRP channels can be activated by diverse stimuli ranging from temperature, mechanical or osmotic stress, chemical compounds, and redox modification (Sawamura et al., 2017; Samanta et al., 2018). Based on sequence homology, the TRP superfamily is divided into six subfamilies in mammals: TRPC (classical or canonical; seven sub-members), TRPM (melastatin; eight sub-members), TRPV (vanilloid; six sub-members), TRPA (ankyrin; one sub-member), TRPP (polycystin; three sub-members), and TRPML (mucolipin; three sub-members) (Figure 1). Notably, most TRP channels (except TRPM4 and TRPM5) are non-selective channels with consistent Ca^2+^ permeability (Guinamard et al., 2011). TRP channels are tetrameric protein complexes that can be assembled into homomeric or heteromeric channels, either with the same subfamily members or with the other subfamily members. Thus, when TRP channels assemble with different subunits, further heterogeneity diversifies their functions. In addition to the physiological roles of TRP channels in neurons, a number of studies regarding the pathological functions have been reported. Intracellular Ca^2+^ influx through TRP channels is involved either in neuronal survival or death and is discussed with respect to the different TRP channel families in the following sections (Bollimuntha et al., 2011).

### TRPC (Classical or Canonical)

TRPC was the first group of TRPs to be discovered in a mammal (Wes et al., 1995), and it shows the highest amino acid similarity to the *Drosophila* TRP channel. The TRPC subfamily is divided into seven subtypes, namely, TRPC1–7. Depending on amino acid similarities, the subtypes are divided into four groups: TRPC1, 2, 3/6/7, 4/5 (Venkatachalam and Montell, 2007). There is still disagreement over the mechanism of action of TRPC; TRPC has been reported to be involved in ion permeation as receptor operated channel (ROC) or to influence intracellular mechanisms of store-operated calcium entry (SOCE) (Vazquez et al., 2004). Recently, as the TRPC channel has been found to have regulation, structure, and novel small molecular probes, research is being actively conducted on it as a therapeutic target for various diseases (Wang et al., 2020).

#### TRPC1

**FIGURE 2:**
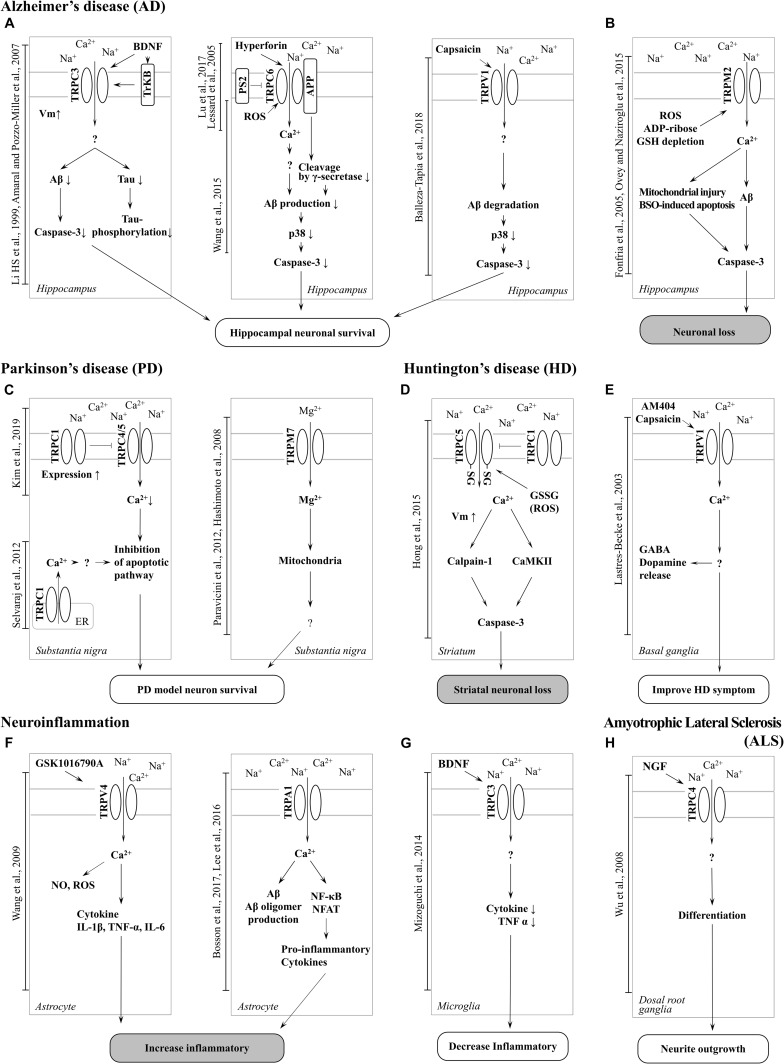
Schematic of TRP channel-mediated mechanisms in neurodegenerative diseases. **(A)** Activation of TRPC3, TRPC6 and TRPV1 channel increase neuronal survival in AD. **(B)** Neuronal loss can be induced by Aβ toxicity, ROS generation, and mitochondrial damage resulting from TRPM2 channel-mediated Ca^2 +^ entry in AD. TRPA1 is also involved in neuroinflammation in AD. **(C)** Inhibition of TRPC4/5 by TRPC1 contribute to inhibition of apoptotic pathways and TRPM7-mediated Mg^2 +^ influx is involved in neuronal survival in PD. **(D)** Increased activity of TRPC5 by oxidative stress induces striatal neuronal loss via Ca^2 +^-dependent pathways in HD. **(E)** Activation of TRPV1 by an agonist improves HD symptoms. **(F)** Activation of TRPV4 and TRPA1 induces a proinflammatory response in astrocytes **(G)** whereas upregulation of surface TRPC3 induced by BDNF regulates microglial functions and reduces inflammation. **(H)** Upregulation of TRPC4 promotes neurite outgrowth and differentiation in DRG (GTEx Consortium, 2013).

In particular, there has been debate about the role or opening mechanisms of TRPC1. Initially, TRPC1 was claimed to take the role of a SOCE in regulating Orai1-mediated Ca^2+^ entry (Ambudkar et al., 2017). Consistent with this claim, the role of TRPC1 in AD has been reported by Linde et al. (2011). Knock-down (KD) of the amyloid precursor protein (APP) gene decreased store-operated Ca^2+^ channel-mediated Ca^2+^ entry and expression of TRPC1 and Orai1 in cultured astrocytes. However, overexpression of APP in TG5469 did not alter TRPC1/4/5 and stored Ca^2+^ level in astrocytes. In SH-SY5Y human neuroblastoma cells, TRPC1 has been reported to reduce expression levels by MPP^+^ (Bollimuntha et al., 2006). Activation of TRPC1 by TRPC1 overexpression or by ER depletion using thapsigargin (TG) ameliorates neurotoxicity. Selvaraj et al. (2009, 2012) showed that Ca^2+^ entry through the activation of store-operated channels (SOC) is important for the survival of dopaminergic neurons (Figure 2C). In the MPTP-induced PD model, TRPC1 expression was suppressed and induced the death of dopaminergic neurons in the substantia nigra. The authors suggested that the cause was reduced interaction with the SOCE modulator stromal interaction molecule 1 (STIM1) and decreased Ca^2+^ entry into the cell. However, our recent study showed that TRPC1 functions as a negative regulator of TRPC4 and TRPC5 (Figure 2C; Kim et al., 2019). Heterodimers of TRPC1/4 and TRPC1/5 suppressed inward current, which may reduce Ca^2+^ influx and Ca^2+^-dependent apoptosis in neurons. We identified that the expression level of endogenous TRPC1 in striatal cells of the HD model was decreased compared to wild-type cells, indicating that HD cells could be more susceptible to oxidative stress due to the activity of the dominant homomeric TRPC5 (Figure 2D; Hong et al., 2015).

#### TRPC3

The important roles of TRPC3 in the hippocampus have been implicated in ND more than in other TRP channels with higher expression levels (Neuner et al., 2015). TRPC3 notably contributes to the maintenance of Ca^2+^ homeostasis and cell growth, such as differentiation and proliferation. In a study conducted by Wu et al. (2004) it was reported that switching proliferation to differentiation is related to TRPC3-induced Ca^2+^ influx and TRPC3-mediated SOCE in the H19-7 hippocampal cell line. Under cell differentiation conditions, TRPC3 expression and TRPC3-induced SOCE levels were increased. The differentiation was blocked by siRNA KD of TRPC3. In addition, TRPC3 is indirectly activated by BDNF. In a study conducted by Li et al. (1999) TRPC3 was activated by neurotrophin receptor TrkB, which is affected by BDNF. A non-selective cationic current was observed in CA1 pyramidal neurons treated with BDNF, although the current was inhibited by siRNA-mediated TRPC3 KD and TrkB-lgG (Amaral and Pozzo-Miller, 2007). This study suggests that BDNF-induced membrane current is due to stimulation of TRPC3 by TrkB (Figure 2A).

In a recent study by Mizoguchi et al. (2014) TRPC3 was found to be involved in the function of microglia, such as the release of cytokines and nitric oxide (NO). Treatment with BDNF rapidly increased the surface expression levels of TRPC3 in rodent microglial cells. In addition, pre-treatment with BDNF inhibited the release NO-induced tumor necrosis factor α (TNFα), which was rescued by treatment with TRPC3 inhibitor. This report suggests that Ca^2+^ influx and concentration maintenance by TRPC3 plays an important role in the improvement of NDs (Figure 2G).

Another characteristic of TRPC3 related to neurodegeneration is directly activated by oxidative stress. The change in Ca^2+^ influx by TRPC3 is associated with neuronal cell death (Selvaraj et al., 2010). Treatment with tertiary butyl hydroperoxide (tBHP) increased the Na^+^ current in HEK293T cells overexpressing TRPC3. Further, Rosker et al. (2004) reported the increase of Na^+^ influx by TRPC3-regulated Ca^2+^ influx in overexpressed HE293T cells. When the Na^+^ concentration of extracellular solution decreased to 5 mM, Ca^2+^ influx was increased by TRPC3 agonist. In addition, treatment of the inhibitor of the Na^+^/Ca^2+^ exchanger strongly inhibited Ca^2+^ influx but Na^+^ did not. This suggests that Ca^2+^ influx by TRPC3 is accompanied by Na^+^/Ca^2+^ exchange. Pesticides, such as rotenone and paraquat, are neurotoxins that induce PD by increasing intracellular oxidative stress. Moreover, both of these pesticides induce the loss of dopaminergic neurons in the SNpc. In a recent study by Roedding et al., chronic treatment of rotenone and paraquat dose-dependently reduced expression levels of TRPC3 and TRPC3-mediated Ca^2+^ influx in primary rat cortical neurons and astrocytes. In another report, OAG-induced Ca^2+^ transients were inhibited in MPP^+^-treated murine striatal astrocytes, and the same was observed in HEK293 cells overexpressing TRPC3 (Streifel et al., 2014). These studies suggest that an increased Na^+^ influx of TRPC3 due to oxidative stress may reduce Ca^2+^ influx and contribute to the treatment of PD. Inhibition of TRPC3 was shown to depolarize GABA neurons in the substantia nigra pars reticulate (SNpr), which are associated with parkinsonism (Zhou et al., 2008). In summary, AD symptoms are recovered by TRPC3 activation. In a previous study, BDNF protected neurons from the neurotoxicity of Aβ and tau (Arancibia et al., 2008; Jiao et al., 2016). TRPC3 activation may decrease Ca^2+^ concentration due to a change in the influx ratio of Na^+^/Ca^2+^. Insufficient Ca^2+^ concentration by TRPC3 activation may be involved in BDNF-induced interference of Aβ plaque formation and tau hyperphosphorylation (Figure 2A).

#### TRPC4 and TRPC5

TRPC4 and TRPC5, which share similar amino acid sequence identity, have important roles in the neuron (Tables 1, 2), in particular with respect to memory in the hippocampus. TRPC5 regulates synaptic plasticity by changing the presynaptic Ca^2+^ homeostasis of hippocampal neurons (Schwarz et al., 2019). TRPC1/4/5 knockout (KO) mice show reduced action potential-triggered excitatory postsynaptic currents (EPSCs) in hippocampal neurons and deficits in spatial working memory (Broker-Lai et al., 2017). However, comprehensive studies of TRPC4 in ND are yet to be undertaken. Only axonal regeneration is associated with TRPC4 expression in the dorsal root ganglia (DRG) (Wu et al., 2008). Neuron growth factor (NGF) and dibutyryl cAMP increase the expression level of TRPC4 in DRG differentiation. Improvement of TRPC4-siRNA reduces the length of neuritis. These results suggest a role for TRPC4 in ALS (Figure 2H). TRPC5, together with TRPC1, has high expression levels in the SN and an important role in dopaminergic neurons (De March et al., 2006). In rat PC12 cells, overexpression of TRPC5 inhibited the neurite outgrowth induced by NGF, and shRNA-mediated KD of TRPC5 enhanced outgrowth (Kumar et al., 2014). TRPC5 has also been reported to regulate neuronal growth cone morphology and nervous system development. In the downstream processes involving semaphorin 3A, growth cone collapse is induced through the cleavage and activation of TRPC5, using calpain (Kaczmarek et al., 2012). In neural progenitor cells, KD of TRPC5 using siRNA reduced the elevation of SOCE and blocked the switch between proliferation and neuronal differentiation (Shin et al., 2010). TRPC5 activity also inhibited neural migration and neurite extension (Tian et al., 2010). Similarly, in the striatum of both YAC128 HD transgenic (Tg) mice and patients, we identified that altered glutathione homeostasis, or increased oxidative potential, resulted in Ca^2+^-dependent apoptosis of striatal neurons, consistent with increased TRPC5 S-glutathionylation and hyperactivation (Figure 2D). Thus, downregulation of TRPC5 activity by siTRPC5 KD and ML204-specific blocker improved the survival of striatal neurons and behavioral motor symptoms (Hong et al., 2015). Furthermore, we recently reported that TRPC5 instability induced by depalmitoylation protects against neuronal death of HD striatal cells (Hong et al., 2019). S-palmitoylation is a reversible covalent lipid modification that promotes membrane trafficking and stability by anchoring the palmitoylated protein to the membrane (Greaves et al., 2009).

**TABLE 1:** Expression levels of TRP channels in human brain, as reported by the Human Protein Atlas.

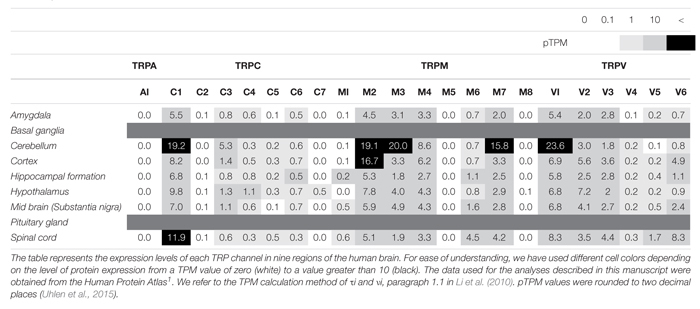

**TABLE 2:** Expression levels of TRP channels in the human brain, as reported by the GTEx project.

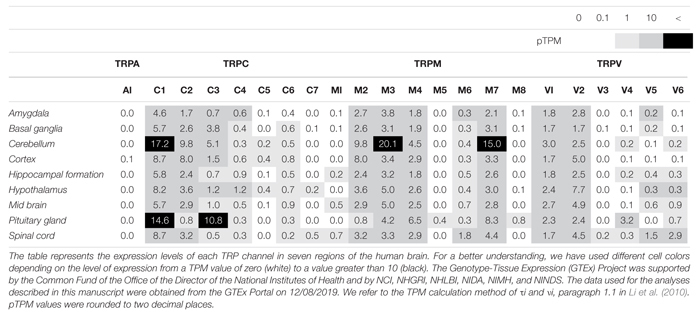

#### TRPC6

It is known that early onset dominant AD is caused by mutations in the APP (Dahlgren et al., 2002) or presenilin 1 (PS1) genes (Dillen and Annaert, 2006). APP is a precursor protein of Aβ, and PS1 is a key enzyme family that cleaves APP in complex with γ-secretase and cleavage of Notch. TRPC6 regulated the mechanism of the PS gene to prevent the progression of AD (Lu et al., 2017). Previous studies have reported that tetrahydrohyperforin, an agonist of TRPC6, lowers Aβ levels and ROS generation, also preventing learning and memory deficits in the AD model. In the research of Dinamarca and colleagues, tetrahydrohyperforin reduced amyloid deposition in rats injected with amyloid fibrils into the hippocampus, inhibited the neurotoxicity of amyloid fibrils and Aβ oligomers in hippocampal neurons, and improved neuropathological behavior in an amyloidosis rat model (Dinamarca et al., 2006). It has also been shown that interaction between TRPC6 and APP leads to inhibition of its cleavage by γ-secretase and a reduction in Aβ production (Wang et al., 2015). The authors also reported that expression of TRPC6 interferes with the interaction of APP (C99) with PS1 but does not interact with Notch. Crossing TRPC6 Tg mice and APP/PS1 model mice reduced plaque load and Aβ levels and improved cognition (Figure 2A). Lessard et al. (2005) reported the effect of PS2 on TRPC6-mediated Ca^2+^ entry. In this study, PS2 inhibited the influx of Ca^2+^ from TRPC6. Induction of Ca^2+^ was higher when FAD-linked PS2, a dominant negative form, was co-expressed with TRPC6 than with wild-type (WT) PS2 and TRPC6 (Figure 2A). However, TRPC6 was still activated by 1-oleoyl-2-acetyl-sn-glycerol (OAG), suggesting that it does not impair channel function^1^.

### TRPM (Melastatin)

The TRPM subfamily has the highest expression level in the brain amongst the TRP channels, and there are many reports in relation to ND. One of the most distinctive features of TRPM ion channels is the high permeability to Ca^2+^ and Mg^2+^ (Hashimoto and Kambe, 2015). The important role of Mg^2+^ signaling in neuroprotection and neurodevelopment has been reported on (Lingam and Robertson, 2018). Thus, studies of TRPM in ND relate pathogenesis to Mg^2+^ signaling.

#### TRPM2

TRPM2 is an ion channel that is abundantly expressed in the brain (Tables 1, 2). TRPM2 has been reported to be activated by a wide range of factors, such as oxidative stress, NAD^+^-related metabolites, and ADP-ribose (Huang et al., 2018). In the study of Belrose et al. (2012) depletion of glutathione (GSH) was reported to be a factor in activating TRPM2. In hippocampal neurons, an increase in ROS due to GSH depletion activated TRPM2, and an increase in TRPM2-dependent Ca^2+^ influx induced neuronal apoptosis (Ovey and Naziroglu, 2015). In the study of Fonfria et al. (2005) an increase in intracellular Ca^2+^ and Aβ induced by TRPM2 activity induced neuronal cell death in rat striatal. Treatment with TRPM2 blocker or SB-750139, which inhibits the production of ADP-ribose, inhibited intracellular Ca^2+^ concentration and cell death via H_2_O_2_ and Aβ. In the study of Ostapchenko et al. (2015) an aged APP/PS1 AD mouse model showed increased ER stress and decreased presynaptic markers (Figure 2B). However, elimination of TRPM2 in APP/PS1 mice improved abnormal response regardless of plaque burden. Age-dependent spatial memory deficits were observed in APP/PS1 mice (Ostapchenko et al., 2015). However, the absence of TRPM2 in these mice attenuated synapse loss and spatial memory. In summary, GSH deficiency and ROS induction activate TRPM2, and Ca^2+^ influx by TRPM2 contributes to the neuronal toxicity of Aβ. TRPM2 may be an important therapeutic target for AD. In the study of PD, Ca^2+^ influx through the TRPM2 channel was induced by ROS and promoted the death of dopaminergic neurons in the SN (Sun et al., 2018). A variant of TRPM2 (P1018L) was found in a Guamanian ALS/PD patient. P1018L attenuates oxidative stress-induced Ca^2+^ influx through TRPM2 (Hermosura et al., 2008).

#### TRPM7

TRPM7 has the Mg^2+^ permeability to maintain the homeostasis of Mg^2+^. In HEK293 cells overexpressing TRPM7, H_2_O_2_ increased Ca^2+^ concentration and TRPM7 current (Nadler et al., 2001). In mouse cortical neurons, TRPM7-siRNA KD and treatment with TRPM7 inhibitors protected against neuronal cell damage (Coombes et al., 2011). In contrast, TRPM7-overexpressing HEK293 cells aggravated cell damage from H_2_O_2_, which was independent of the voltage-gated Ca^2+^ channel. Interestingly, in the study of Aarts et al. (2003) blocking of Ca^2+^-permeable non-selective cation conductance or KD of TRPM7 inhibited TRPM7 currents, anoxic Ca^2+^ uptake, ROS production, and anoxic death in cortical neurons. Mg^2+^ permeability of TRPM7 is implicated in PD (Figure 2C). Continuous administration of Mg^2+^ significantly inhibited the neurotoxicity of MPP^+^, reduced the death of dopaminergic neurons, and improved the length of dopaminergic neurites (Hashimoto et al., 2008). In a recent zebrafish study, TRPM7 mutation suppressed dopamine-dependent developmental transitions and increased sensitivity to the neurotoxicity of MPP^+^ (Decker et al., 2014), and expression of the channel-dead variant of TRPM7 in SH-SY5Y cells increased cell death. These studies suggest that the role of Mg^2+^ influx and TRPM7 in dopaminergic neurons is important and could be a therapeutic target for PD. A variant of TRPM7 (T1482I) was also found in Guamanian ALS/PD cases. Incidentally, mutant G93A-superoxide dismutase (SOD1) mice are used as an ALS model (Guo et al., 2009).

### TRPV (Vanilloid)

The TRPV subfamily has been reported to have the highest number of sensory functions, such as nociception, mechano-sensing, osmolarity-sensing, and thermo-sensing. Usually, TRPV is expressed in peripheral sensory nerves, although pathological studies have also reported expression in the brain. The various antagonists of TRPV4 could protect damaged neurons and inhibit the production of ROS (Suresh et al., 2018; Wu et al., 2019).

#### TRPV1

TRPV1 is expressed not only in the plasma membrane but also in the ER and calcium storage vesicles (Marshall et al., 2003). TRPV1 is phosphorylated to enable translocation from the ER to the plasma membrane. TrkA activity due to NGF increases the surface expression level of TRPV1 located in the ER through PKC phosphorylation (Zhang et al., 2005). An increase in intracellular calcium levels due to TRPV1 activity may aggravate neuronal cell death. In microglia cells, TRPV1 activity by agonists such as capsaicin (CAP) and resiniferatoxin (RTX) induce apoptosis (Kim et al., 2006). Dopamine release is dependent on the mechanosensitive TRPV1 channels activated by cannabinoid receptor stimulation in dopaminergic neurons (Oakes et al., 2019). Capsaicin, a TRPV1 agonist, induces the death of mesencephalic dopaminergic neurons through the activation of TRPV1 and CB1 receptors. Activation of TRPV1 increases the release of the mitochondrial cytochrome C and caspase3 cleavage. Cell damage is attenuated by an intracellular Ca^2+^-chelator. In a recent study, the activity of TRPV1 was reported to decrease Aβ-induced cytotoxicity (Balleza-Tapia et al., 2018). Treatment with a TRPV1 agonist rescued Aβ-induced degradation of hippocampal neuron function (Figure 2A). In addition, it is suggested that TRPV1 contributes to the movement of patients with HD (Figure 2E). In the 3-nitropropionic acid-induced HD model, hyperkinesia was attenuated by administering AM404, an endocannabinoid reuptake inhibitor (Lastres-Becker et al., 2003). This phenomenon is reversed by the TRPV1 antagonist capsazepine, suggesting that TRPV1 activity may facilitate the movement of HD patients. Depending on the pathological mechanism, the role of TRPV1 activity can be distinguished accordingly and can be an important drug development target of NDs.

#### TRPV4

The activity of TRPV4 causes neuronal injury in pathological conditions. In many types of cells, TRPV4 activity increases the production of ROS and NO (Hong et al., 2016). GSK1016790A, an agonist of TRPV4, increased the concentration and NO in the hippocampus. TRPV4 agonist-induced neuronal cell death in hippocampal CA1 was inhibited by treatment with ROS scavengers such as Trolox or ARL-17477. In a recent report, TRPV4 enhanced neuronal inflammatory responses and pro-inflammatory cytokine release (Figure 2F; Wang et al., 2019). GSK1016790A-injected mice also showed increased levels of the pro-inflammatory cytokines IL-1β, TNF-α, and IL-6 and showed TRPV4-mediated microglial and astrocyte activation. Although direct evidence linking TRPV4 to NDs has not been reported, these results suggest a clear association between neuronal cell death and ROS.

### TRPA

The TRP ankyrin 1 (TRPA1) channel is a non-selective transmembrane cation channel with multiple ankyrin repeats at its N-terminal. TRPA1 is mainly expressed in primary sensory neurons and non-neuronal cells (Jo et al., 2013). According to RNA-seq ALTAS data (Tables 1, 2), the expression level of TRPA1 in the brain is low, but various functions have been reported in recent studies. Reported TRPA1 functions are mainly the detection of pain, cold temperature, and cannabinoids, in addition to noxious compounds that elicit pain and neurogenic inflammation (Paulsen et al., 2015).

#### TRPA1

Until now, the role of TRPA1 in neurons has only been reported on with respect to pain and inflammation, although recent studies have revealed a potential involvement in AD pathogenesis. Deposition of Aβ is an important factor in the exacerbation of AD, and soluble Aβ oligomers mediate fast and widespread Ca^2+^ influx in astrocytes (Bosson et al., 2017). TRPA1 was first identified in mouse hippocampal astrocytes and associated with Aβ-mediated Ca^2+^ signaling (Figure 2F; Lee et al., 2016). The cause of Aβ oligomer-mediated fast Ca^2+^ signaling appears to be the hyperactivation of TRPA1 (Bosson et al., 2017). TRPA1-induced Ca^2+^ signaling initiates the release of inflammatory factors such as PP2B, NF-κB, and NFAT (Figure 2F). APP/PS1 Tg mice, an AD mouse model, have increased expression of TRPA1 in hippocampal astrocytes. Loss of function of TRPA1 channels improves spatial learning, memory and cognition, and decreases Aβ deposition in APP/PS1 Tg mouse also (Lee et al., 2016). In summary, TRPA1-induced Ca^2+^ influx in astrocytes may be evidence of the critical role of Aβ in inflammatory processes and AD progression. Drug development focused on TRPA1 could be a novel target for treating dysfunction in AD.

## TRP Channels: Novel Therapeutic Candidate for Neurodegenerative Disease

Various causes of NDs have been reported recently. However, most of the brain lesions in ND present alongside several pathological environments, such as the presence of ROS, impaired antioxidant systems, and disrupted Ca^2+^ homeostasis (Di Meo et al., 2016). In consideration of the results discussed above, the regulation of TRP channels plays a key role in the Ca^2+^-dependent neuronal death in NDs. The involvement of TRP channels in NDs is summarized in Figure 1. To investigate the role of TRP channels in NDs, TRP channel antagonists and TRP-KO mice have been generated and utilized. TRP KO mice exhibit behavioral and neurological phenotypes.

TRPC3 is required for slow excitatory postsynaptic potential in cerebellar Purkinje cell synapses, and consequently, severe ataxic phenotypes have been shown in TRPC3 KO mice. In contrast to TRPC1/4 double-KO or TRPC1/4/6 triple-KO mice, TRPC3 KO mice show movement deficits of the hind-paws (Hartmann et al., 2008). In addition, TRPC4 plays a role in fear and anxiety-related behaviors. TRPC4 KO mice show innate fear responses in elevated plus maze and open-field tests. These fear responses result from mGluR-mediated EPSC in the lateral nucleus of the amygdala neurons (Riccio et al., 2014). TRPC5 also plays an essential role in fear-related behavior. In addition, disruption of burst firing in the potent muscarinic antagonist pilocarpine-induced seizure in TRPC5 KO mice was reduced. Seizure-induced neuronal loss in the hippocampal region was also reduced in TRPC5 KO mice (Phelan et al., 2013). TRPM2 KO mice exhibited disturbed EEG rhythms and bipolar disease-related behavior, including impairment of social behavior and increased anxiety (Jang et al., 2015). TRPM7 KO mice exhibited clasping, tremors, and slow movement associated with Mg^2+^ deficiency (Ryazanova et al., 2010).

TRPC KO mice combined with ND models have also been generated. In models of AD, TRPC6 modulates cleavage of APP by gamma secretase and APP (C99) interaction with PS1 (Wang et al., 2015). TRPC6 overexpression in APP/PS1 mice results in a reduction of Aβ accumulation in the hippocampus (Table 3). Therefore, TRPPC6 overexpression improves spatial learning and memory in APP/PS1 mice. In addition, the expression of the inflammatory factors TNF-α, IL-1β, COX-2, and IL-6 is regulated by levels of TRPC6 via Aβ, and levels of TRPC6 are increased by Aβ via NF-κB in BV-2 microglia cells (Lu et al., 2018). TRPM2 expression is involved in synapse loss, microglial activation, and spatial memory deficits in APP/PS1 mice (Ostapchenko et al., 2015). Activation of TRPV1 channels is required to trigger long-term depression at interneuronal synapses (Gibson et al., 2008) and prevents Aβ-involved impairment of functional networks in the hippocampus (Balleza-Tapia et al., 2018). Astrocytic Ca^2+^ hyperactivity is induced by Aβ oligomers via TRPA1 in the hippocampus. Moreover, astrocyte hyper-excitability is replaced by CA1 neuronal activity in APP/PS1 mice (Lee et al., 2016). Moreover, TRPA1 regulates astrocyte-derived inflammation in APP/PS1 mice. TRP channel antagonists regulate the production of ROS, APP processing, and Aβ accumulation. The TRPV4 antagonist HC-067047 attenuates the H_2_O_2_-induced Ca^2+^ influx (Suresh et al., 2015). Aβ-mediated cell damage was attenuated by treatment with TRPV4 blockers ruthenium red and gadolinium chloride (Bai and Lipski, 2014).

**TABLE 3:** Disease-related functions of TRP channels.

***Disease***	***Region***	***Channel***		***Mechanism of related disease***	***References***
**NDs**	Microglia	TRPC	C3	Inhibit to release cytokines and NO	Mizoguchi et al., 2014
	Neuronal progenitor		C5	Reduce elevation of SOCE Regulate the switching between proliferation and differentiation	Shin et al., 2010
	Hippocampus	TRPM	M2	Activate due to ROS and increase Ca^2+^-mediated cell death	Ovey and Naziroglu, 2015
	Cortical		M7	Aggravate cell damage by increase Ca^2+^ induced oxidative stress	Coombes et al., 2011
	Microglia	TRPV	V1	Increase neuronal cell death by agonist, such as cannabinoid, Capsaicin	Kim et al., 2005
	Microglia Astrocyte		V4	Enhance neuronal inflammatory responses Inhibit pro-inflammatory cytokine release	Wang et al., 2019
	Hippocampus	TRPC	C3	Regulate to switch between proliferation and differentiation Change influx ratio of Na^+^/Ca^2+^ when activate by oxidative stress	Wu et al., 2004; Rosker et al., 2004
			C5	Regulate neuronal growth cone morphology and nervous system development	Kumar et al., 2014; Kaczmarek et al., 2012
			C6	Inhibit function of y-secretase and reduced Aβ level by PS1 PS2 regulate TRPC6-mediated Ca^2+^ entry Interaction between TRPC6 and APP inhibit PS1 process	Lu et al., 2017; Lessard et al., 2005; Wang et al., 2015
**AD**	Hippocampus Striatum	TRPM	M2	Increase Aβ-mediated and Ca^2+^-mediated cell death Damage to neuronal cell in APP/PS mice	[Bibr B37][Bibr B90]
	Hippocampus	TRPV	V1	Decrease Aβ-induced cytotoxicity and apoptosis	Balleza-Tapia et al., 2018
			V4	Aggravate neuronal cell death from oxidative stress	Hong et al., 2016
		TRPA	A1	Exacerbate spatial learning, memory and cognition Increase Aβ deposition and release inflammatory factors	Lee et al., 2016
**PD**	Substantia nigra	TRPC	C1	Decrease neurotoxicity and unfolded protein response Regulate SOCE and increase survival of dopaminergic neuron	Bollimuntha et al., 2006 Selvaraj et al., 2009, 2012
	Dopaminergic neuron	TRPM	M7	Reduce neurons death and activate growth the length of DA neurites	Hashimoto et al., 2008; Oakes et al., 2019
**HD**	Striatum	TRPC	C1	Inhibit neuronal cell death by reducing TRPC5 activity	Hong et al., 2015
			C5	Increase neuronal apoptosis by activation induced oxidative stress	Hong et al., 2015
	Basal ganglia	TRPV	V1	Improve the movement of HD patient models	Lastres-Becker et al., 2003
**ALS**	DRG	TRPC	C4	Activate growth of neurite length and regulation of differentiation	Wu et al., 2008

*ND, neurodegenerative diseases; AD, Alzheimer’s disease; PD, Parkinson’s disease; HD, Huntington’s disease; ALS, amyotrophic lateral sclerosis; SOCE, store-operated calcium entry; PS, presenilin1; ROS, reactive oxygen species; Aβ, amyloid-beta; DA, dopamine; DRG, dorsal root ganglia.*

TRPC1, TRPC3, TRPM2, TRPM7, and TRPV1 have been shown to be involved in PD. TRPC1 activation reduces dopaminergic neuronal death (Selvaraj et al., 2009). TRPC3-mediated Ca^2+^ influx contributes to the survival of neurons in the SN (Zhou et al., 2008). Additionally, MPP^+^-induced oxidative stress increases intracellular Ca^2+^ via TRPM2 activation (Sun et al., 2018). TRPM7 channels regulate magnesium homeostasis in cells, and the presence of Mg ameliorates MPP^+^ toxicity (Hashimoto et al., 2008; Paravicini et al., 2012). Ca^2+^ influx via TRPV1 in dopaminergic neurons mediates mitochondrial dysfunction, microglial activation, ROS generation, and cell death (Kim et al., 2005; Nam et al., 2015). TRPV1 or TRPN-like channel-dependent dopamine release is mediated by CB1 stimulation (Table 3; Oakes et al., 2019). In addition, regulation of TRPV1 activity is closely related to the survival of dopaminergic neurons. TRPM2 is controlled by oxidative stress (Belrose et al., 2012; Huang et al., 2018). Therefore, the regulation of TRP channels contributes to overcoming dopamine depletion and the loss of dopaminergic neurons in PD patients. Likewise, oxidizing modulation of posttranslational modification (glutathionylation) of TRPC5 leads to apoptosis in an HD model (Figure 2D; Hong et al., 2015). Attenuation of TRPC5 activity by KD, blocker, or depalmitoylation shows therapeutic effects against oxidative stress by lowering TRPC5 toxicity (Hong et al., 2019). Additionally, regulation of TRPML1, TRPM2, and TRPM7 activity might also be a therapeutic strategy for ALS (Hermosura and Garruto, 2007; Hermosura et al., 2008; Tedeschi et al., 2019). The TRPV1 antagonist capsazepine has antihyperkinetic effects in a model of HD (Lastres-Becker et al., 2003).

As previously reported, TRP channels can be assembled as homo- or heteromeric complexes in nature. However, individual TRP channel KO models that lack phenotypes are limited in their ability to determine the cause of the functional compensation of each TRP channel. Moreover, *in vivo* studies regarding chronological changes in TRP channel expression patterns in the brain are needed for NDs. The regulation of TRP channels can be a novel therapeutic target for NDs. Nevertheless, a limitation to the development of TRP channel-specific antagonists is that their structures remain unknown. Therefore, structural analyses must precede pathological and clinical studies.

## Conclusion

Transient receptor potential channels may not be the only, or main, pathogenic factors contributing to the pathogenesis of ND. Research in the field of ND is challenging; it is necessary to either conclusively prove a relationship between pathogenic factors or identify new therapeutic targets. In achieving one of these two possibilities, it is important not to underestimate the potential of TRP channels, based on the physiological and pathological functions of TRP channels discovered so far. Through the interrelationship between disruption of Ca^2+^ homeostasis and the development of NDs, lowering the activity of TRP channels is sufficient to enable expectations for new therapeutic strategies. As we have discussed, increased TRP channel activity has been widely observed in NDs, and model studies have shown that abnormal function due to upregulation of TRP channels can be controlled by drug treatments. Since many drugs have been reported to be TRP channel inhibitors, understanding binding modes will provide deep insight for pharmacological application in NDs. However, direct evidence for drug binding to TRP channels is unavailable. To address this issue, the most effective approach for understanding drug binding would be a structural study.

## Possibilities of Drug-Bound Structure

For several decades, the detailed structures and topologies of TRP channels were not understood, although the first high-resolution structure of TRPV1 was recently resolved by a single particle cryo-EM (Liao et al., 2013). Since TRPV1 was the first membrane protein to be characterized from single particle cryo-EM and the biochemical methods were relatively similar for other family members, most follow-up studies concentrated on TRP channel structure. However, though high-resolution structures have been resolved for most ND-related TRP channels, the structures of the drug-bound forms are mostly unknown. Here we discuss the technical possibilities for determining the drug-bound structure of the TRP channel family.

For analysis of drug-channel binding, which would improve our understanding of the inhibition mechanisms and would provide clues for developing drug design, the determination of the high-resolution structure is absolutely necessary. In the TRP family, the resolutions of most ND-related TRP structures are above 3.5 Å, with a few exceptions such as TRPC6, TRPA1, and TRPV4 (3.8, 4.24, and 3.8 Å, respectively) (Paulsen et al., 2015; Deng et al., 2018; Tang et al., 2018). TRPC5 broke the 3 Å barrier (Duan et al., 2019), and TRPM2 also came close to hitting the barrier (3.07 Å) (Zhang et al., 2018). The structures of other members of the TRP family that are associated with NDs, such as TRPC3, TRPC4, TRPM7, and TRPV1, were determined at ∼3.3 Å (Liao et al., 2013; Duan et al., 2018; Fan et al., 2018; Liu et al., 2018). Therefore, there is still room for improvement in their resolutions, possibly by biochemical techniques such as nanodisc reconstruction. The resolution of the drug-bound form does not always guarantee higher resolution than that of the apo structure, but generally, the occupation of an inhibitor in the binding cavity facilitates conformational stabilization, resulting in higher resolution. Therefore, since there is optimism regarding drug-binding studies, we suggest that ongoing attention and efforts be focused on this area of therapeutic target research.

## Author Contributions

CH, BJ, HP, and IS wrote and discussed the review at almost all stages. JL professionally edited the manuscript for English language. JC, JK, and Y-CS analyzed representative tables presenting TRP channel expression data and also provided technical support. IS and CH supervised the entire writing process.

## Conflict of Interest

The authors declare that the research was conducted in the absence of any commercial or financial relationships that could be construed as a potential conflict of interest.

## Footnotes

1 http://www.proteinatlas.org
